# The role of NK cells in oncolytic viral therapy: a focus on hepatocellular carcinoma

**DOI:** 10.20517/jtgg.2021.27

**Published:** 2021-08-04

**Authors:** Frazer Warricker, Salim I. Khakoo, Matthew D. Blunt

**Affiliations:** Clinical and Experimental Sciences Unit, University of Southampton, Southampton SO16 6YD, UK

**Keywords:** Oncolytic virus, natural killer cell, NK cell, immunotherapy, hepatocellular carcinoma

## Abstract

Natural killer (NK) cells have a key role in host anti-tumour immune responses via direct killing of tumour cells and promotion of adaptive immune responses. They are therefore attractive targets to promote the anti-tumour efficacy of oncolytic viral therapies. However, NK cells are also potent components of the host anti-viral immune response, and therefore have the potential for detrimental anti-viral responses, limiting the spread and persistence of oncolytic viruses. Oncolytic viruses are currently being investigated for the treatment of hepatocellular carcinoma (HCC), a leading cause of cancer-related death with a high unmet clinical need. In this review, we highlight the role of NK cells in oncolytic virus therapy, their potential for improving treatment options for patients with HCC, and discuss current and potential strategies targeting NK cells in combination with oncolytic viral therapies.

## Introduction

### NK cells

Discovered in 1975, natural killer (NK) cells are a subset of innate lymphocytes that play an important role in anti-tumour and anti-viral responses. In humans, NK cells are characterised as CD3^-^CD16^+^CD56^+^ lymphocytes that reside in lymphoid (bone marrow and lymph nodes) and non-lymphoid tissues (liver, gut, lung, and skin) and do not require priming to initiate effector responses^[1]^. NK cells detect virally infected or transformed cells via an array of germline-encoded non-rearranging activating and inhibitory receptors^[2,3]^. They are cytotoxic in nature releasing granules containing cytolytic molecules (granzyme B, perforin) that induce death in infected or stressed cells^[4]^. Furthermore, they possess the ability to secrete an array of cytokines, chemokines, and growth factors to co-ordinate an adaptive immune response through cross-talk with other immune cells, including dendritic cells and T cells^[5]^.

Growing evidence highlights the crucial role of NK cells in the anti-tumour response with their population size, function, and localisation positively correlating with survival in patients with various cancers^[6]^. NK cells have been implicated in the immune response against hepatocellular carcinoma (HCC). For example, patients with HCC have a significant reduction in peripheral CD56^dim^CD16^+^ NK cell populations compared to healthy controls^[7]^. Additionally, within the intra-tumoral tissue of HCC, there may be low levels of NK cell infiltration compared to their levels within the adjacent liver tissue^[7]^. Furthermore, a positive relationship has been identified between the density of intra-tumoral NK cells and overall survival in HCC patients^[8]^. NK cells have been found to be dysfunctional within the tumour microenvironment (TME) of HCC with a marked reduction in interferon-gamma (IFN-γ) and TNF-α production in both the intra-tumoral and peripheral NK cell populations compared to healthy individuals^[7,9]^. This blunted cytokine production may be secondary to inhibitory cells such as tumour-associated monocytes with high levels of CD48 expression that contribute to NK cell dysfunction through interaction with CD244 (2B4) on the NK cell surface^[9]^. Understanding the role NK cells play not only in the direct killing of tumour cells, but also in the modulation of the TME and potentiation of adaptive immunity is likely key for the design of successful NK cell immunotherapies.

## Introduction to Oncolytic Virus Therapies

Oncolytic viruses (OV) are therapeutically useful viruses that can preferentially replicate and kill cancer cells whilst being relatively non-pathogenic to healthy human cells. They can be based on both DNA viruses (e.g., pox virus, vaccina virus and adenovirus) and RNA viruses (e.g., reovirus and picorna viruses). To fully optimise OV therapy, the virus must efficiently infect and lyse tumour cells, and potentially stimulate a potent systemic anti-tumour immune response^[10]^.

Only three OVs have been approved for market use. Approximately 100 clinical trials relating to OV therapy have been completed to date, but only two have been phase III trials^[11,12]^. The first to come to market was Rigvir, a non-pathogenic ECHO-7 picornavirus used in the treatment of melanoma. Since emerging on the market in 2004, its use has been restricted to Latvia, Georgia, and Armenia, with the limited available evidence of benefit being that of improved survival in patients with early-stage disease^[13–15]^.

Oncorine was the first recombinant OV approved in 2005 in patients with head and neck cancer in China. It is an adenovirus with deletions in viral E1B-55k and E3, allowing infection and replication within p53 positive tumours^[16,17]^. Exciting results were seen in initial clinical studies where response rates with Oncorine plus chemotherapy (cisplatin and 5-fuorouracil) compared to chemotherapy alone were 79.8% and 39.6% respectively^[18]^. However, a drawback of this therapeutic is the significant baseline seroprevalence of antibodies against Adenovirus Serotype 5, which forms the core of Oncorine, thus limiting its use to intravenous therapy in metastatic disease. Adenoviral vectors using viruses with a lower seroprevalence are currently in development to circumvent this issue.

The third approved OV is talimogene laherparepvec (T-VEC). It was approved by the United States FDA in 2015 for non-resectable metastatic melanoma and licensed in Europe for locally advanced cutaneous melanoma. T-VEC is a recombinant herpes simplex virus type 1 (HSV-1) and encodes two copies of granulocyte-macrophage colony-stimulating factor using CMV-promoters. T-VEC can be used as a standalone treatment or combined with the PD-1 inhibitor pembrolizumab or the CTLA-4 inhibitor ipilimumab^[19,20]^.

## Effect of the Oncolytic Virus on Immune Cells

The chief goals of OV therapy are replication-dependent oncolysis and activation of the immune system, although the TME can now also be considered a relevant therapeutic target^[21]^. Both the innate and adaptive arms of the immune response are modulated by OV therapy. For example, after infecting cancer cells, an inflammatory reaction occurs when OV initiates immunogenic cell death. It stimulates an anti-tumour response through the activation and recruitment of dendritic cells exposed to released damage-associated molecular patterns (DAMPs)^[22]^. DAMPs and other metabolites, including tumour-associated and tumourspecific antigens, are recognised by antigen-presenting cells (APC) within the TME. These generate virus and tumour-specific T cells, and antibodies to propagate systemic and long-term anti-tumour responses, which may be beneficial in both local and metastatic disease^[23]^. Thus, the primary immune response from OV infection consists of cytokine release and modulating the TME from immunologically “cold” and immunotolerant, to “hot” and immunoreactive.

While triggering an anti-cancer immune response, an unwanted side effect is the generation of anti-viral responses. These may induce premature clearance of OV and thus limit its effectiveness. Conversely, the anti-viral response is important in triggering the initial anti-cancer response, with T cells lysing tumour cells containing viral antigens^[24]^. It highlights the importance of engineering OVs with the capacity to quickly replicate and infiltrate tumours and so optimise the host anti-tumour response prior to the clearance of viral particles. Repeated local intra-tumour injections have been found to improve anti-cancer efficacy in animal models. For instance, Li *et al*.^[24]^ found that in a pancreatic cancer model treated with adenovirus, adaptive immunity plays a relatively more important role than direct tumour lysis when administering OV into the tumour.

## Innate Immune Cells and OV Therapy

Owing to the nature of OV therapy, there is a limited window for therapeutic efficacy to occur, implying that innate immune responses are likely to be important. The ideal OV is one that maximises the anticancer immune response whilst minimising responses against the OV^[25]^. OVs can be administered peripherally and can reach distant lesions. However, a drawback of this approach is the increased exposure of the OV to the host immune system, making the OV vulnerable to clearance. Macrophages are scavenger cells that act as the first barrier. They directly recognise pathogens initiating an inflammatory response, and systemic delivery exposes the OV to the risk of macrophage phagocytosis^[26,27]^. To address this challenge, viral coat proteins have been modified using polyethene glycol to prevent recognition of the virus and improve its access to the tumour^[28]^. In addition to the release of anti-viral cytokines, innate immune cells possess mechanisms that can aid anti-tumour efficacy of OV therapy. In response to OV therapy, neutrophils can accumulate in the tumour, destroying the tumour vasculature and inducing apoptosis through secretion of reactive oxygen species, cytokines and proteases, including CXCL1 and CXCL5^[29,30]^.

NK cells are also key players in anti-tumour responses following OV therapy^[31,32]^. NK cells target cells lacking MHC molecules or expressing markers of cellular stress and upon activation release cytotoxic granules, chemokines and cytokines, culminating in apoptosis of the target cells and promotion of adaptive immunity^[33,34]^. NK cell activation and participation in tumour clearance following OV infection have been shown for various OV strains, including herpes simplex virus^[35]^, measles virus^[36]^, vesicular stomatitis virus^[37]^, Newcastle disease virus^[38]^ and myxoma virus^[39]^. NK cells enhanced the cytotoxicity of oncolytic adenovirus against infected cells, and interestingly, NK cells activated by oncolytic adenovirus also showed enhanced cytotoxicity against non-infected tumour cells^[40]^. Importantly, cell depletion studies *in vivo* have revealed the crucial role of NK cells in mediating efficacy of multiple oncolytic viruses via their direct cytotoxicity and cytokine production^[41,42]^. Furthermore, in an HCC model, oncolytic reovirus therapy induced significant NK cell infiltration of the tumour^[43]^. In this model, type 1 interferon was a key determinant in indirectly initiating the degranulation of NK cells and activation of NK cells was required for anti-tumour responses^[43]^. The advantage of simultaneous activation of NK cells in combination with OV therapy includes the promotion of cytotoxicity and augmentation of an adaptive immune response. NK cells detect tumour cells via a plethora of receptors and can kill non-infected and MHC-I downregulated tumour cells^[40]^. In addition to their direct cytolytic function, NK cells are required for optimal cytotoxic T cell responses against cancer via the recruitment and maturation of dendritic cells in the tumour bed^[6,44]^. NK cells also produce IFN-γ in the TME, which upregulates MHC-I expression by tumour cells^[45]^, thereby increasing the presentation of neoantigens to cytotoxic T cells. In addition, NK depleted tumours have reduced IFN-γ and TNF-α expressing T cells^[46]^. Therefore, targeting NK cells in combination with T cells is thought to be required for sustained tumour regression. As key anti-viral immune cells, however, NK cell activation can also be detrimental to OV persistence and spread and this is discussed further below.

## Adaptive Immunity and OV

Presentation of viral or tumour associated antigens to adaptive immune cells triggers a specific immune response. It predominantly involves the cytotoxic T lymphocyte (CTL). They recognise specific antigens on MHC class I molecules on the surface of infected and malignant cells. Following recognition, they induce cell death through apoptosis and the release of cytotoxic granules similar to NK cells. Work has shown that CTLs specific to viral antigens appears first, and those specific for tumour-associated antigens follow^[24]^. Helper T cell & B cell responses are also generated, leading to a robust immune response, which may be both beneficial in clearing tumours, but detrimental in accelerating the clearance of OVs. It is increasingly becoming appreciated that NK cells also have an important role in the promotion of the adaptive immune response via the promotion of dendritic cells and CD8^+^ T cell recruitment into tumours^[6,44]^. The simultaneous activation of NK cells and T cells may therefore promote long-term anti-tumour immunity and a more beneficial anti-tumour immune response.

## Anti-Viral Immune Response and Detrimental Clearance of Oncolytic Virus

OV spread and persistence are crucial for the activation of anti-tumour immune responses, and the simultaneous activation of anti-viral immune responses can negatively affect therapeutic efficacy. Because OVs are largely selected for their ability to infect human cells, patients may already be primed for an antiviral immune response against the particular OV used through prior exposure to similar viruses during either natural infection or vaccination.

Immune effector populations which mediate anti-viral immunity include tumour-associated macrophages, which engulf and destroy virally infected cells. These T cells produce cytokines to orchestrate an anti-viral immune response and B cell-mediated production of virus-neutralising antibodies (reviewed by Lemos de Matos *et al*.^[25]^). In particular virus-neutralising antibodies may limit OV transduction efficiency^[47]^, and consistent with this low pre-treatment, anti-viral neutralising antibody levels are associated with a more successful outcome following OV therapy^[48]^.

As NK cells are the first-line of defence against viral infections, they can play a role in the limitation of OV spread and persistence. The ability of NK cells to clear OV virus *in vivo*, however, appears to differ depending on the viral strain or tumour model used. For example, NK cells have been shown to limit the efficacy of oncolytic HSV-1 in a murine model of glioblastoma^[49]^. In this model, NK cells were recruited to the OV infection site within hours and orchestrated an anti-OV effect through direct cytotoxicity against tumour cells (dependent on NKp30 and NKp46) and through IFN-γ dependent activation of microglia and macrophages within tumours^[49]^. By contrast, NK cell recruitment and activation were associated with promoting anti-tumour effects following injection of an IL-12 expressing Maraba virus vaccine against peritoneal tumours^[50]^ with increased IFN-γ, and granzyme B production, NK mediated cytotoxicity and migration^[50]^. Importantly, in colorectal cancer patients with liver metastases, NK cell activation was evident following oncolytic reovirus treatment^[51]^, and pre-clinical models have shown that oncolytic reovirus induces NK cell cytotoxicity against tumour cells^[52]^. Therefore, it is important to clarify the role of NK cells in viral clearance for each viral strain and tumour type to understand whether NK cells will be beneficial or detrimental to a specific OV therapy in patients. To this end transient, NK depletion prior to OV therapy and subsequent NK therapy following OV therapy was considered to provide optimal benefit in a treatment combing OV therapy with the proteasome inhibitor bortezomib^[53]^. Using a combination of mathematical modelling and *in vivo* experimentation, the authors showed that both depletion of NK cells prior to OV and infusion of NK cells after OV therapy increased the survival of tumour-bearing mice. However, given the risks of infection associated with depletion of NK cells, it may be clinically advantageous to focus on augmenting NK cell function after OV therapy rather than inhibiting them prior to OV therapy.

An alternative approach under evaluation to overcome activation of anti-viral immune responses to improve OV distribution/persistence is the modification of the OV itself. For example, oncolytic adenovirus armed with the IL-24 gene and coated with amphiphilic phospholipids and calcium phosphate (PLC-ZD55-IL-24) reduced OV sequestration in the liver, reduced innate immune cell activation and also protected the OV from pre-existing neutralising antibodies compared to unmodified virus (ZD55-IL-24)^[54]^. It enhanced *in vivo* anti-tumour effects of PLC-ZD55-IL-24 compared to uncoated ZD55-IL-24 virus against the liver cancer-derived cell line Huh7^[54]^. It is clear that NK cells play a complex role in OV therapy, interplaying beneficial anti-tumour immunity and detrimental anti-viral immunity. As such, strategies to promote NK cell activity alongside OV therapy must take this into consideration to ensure maximal therapeutic benefit.

## Hepatocellular Carcinoma

Worldwide, liver cancer is the third leading cause of cancer-related death with the fifth-highest incidence (Globoscan 2020, WHO factsheet). Unfortunately, the incidence of HCC is still on the rise, with the United States seeing a 40% increase in the rate of HCC death from 1990 to 2004, illustrating the critical requirement for new therapeutic options^[55]^. HCC is the most prevalent form of primary liver cancer with a propensity to affect males more than females. Hepatitis B (HBV) and Hepatitis C virus (HCV) are the 2 most common causes of HCC, accounting for 80% of cases globally^[56]^. Other key risk factors include alcohol-related liver disease, non-alcoholic fatty liver disease (NAFLD) and metabolic-related liver conditions. There is a strong correlation between chronic liver disease and HCC, a complication of the natural history of liver cirrhosis^[57]^.

There are significant variations in HCC incidence globally. It is mainly due to the subpopulation’s exposure to relevant environmental and infectious risk factors. The highest incidence is seen in Africa and Asia, where the prevalence of HBV & HCV is greatest^[58]^. Chronic HBV infection is the leading cause of HCC in most African countries, whilst in North America and Europe, HCV is the most frequent virus-related cause of HCC development^[59]^. NAFLD, with a prevalence of approximately 25% globally, is the most common liver disease and represents a significant risk factor for HCC comprising 10%-20% of cases in the United States^[60,61]^. Independent of NAFLD, features of the metabolic syndrome such as obesity and diabetes mellitus confer increased HCC risk^[62,63]^. The global epidemic of obesity is likely to ensure that HCC related to NAFLD continues to increase over the coming years.

As HCC can arise on a diverse background of underlying liver diseases, it is not surprising that it is heterogeneous in nature. HCC has been classified by both its immunological and molecular characteristics^[64,65]^. In general, HCC arising on the background of chronic viral infection are more immunoreactive compared to those arising as a consequence of metabolic disease^[66]^. These factors imply that a stratified approach to HCC is more likely to be successful, especially when taking into account the complex immunology that surrounds a successful OV treatment.

In North America and Europe, more than 60% of patients with HCC present at an intermediate or advanced-stage precluding them from curative therapies such as liver resection or liver transplantation^[59]^. Loco-regional therapy and systemic therapy are the remaining treatment options for advanced HCC, representing about 70% of patients at the time of diagnosis^[67]^. At present 1st line options for systemic treatment include Sorafenib and Lenvatinib, with the combination of Atezolizumab & Bevacizumab (PD-L1 & VEGF inhibitors respectively) an exciting new first-line prospect. Despite the emergence of this new therapy, overall survival remains low at 67% at 12 months, with progression-free survival around 7 months^[68]^. Furthermore, treatments available, including immunotherapy, can be associated with poor tolerability and significant toxicity due to the underlying liver disease^[69]^. Therefore, there is still a critical requirement and ongoing drive for the development of new strategies aiming to provide more targeted and effective therapy for patients with HCC. Due to their major immunoregulatory properties, NK cells have been of interest with varying avenues of immunotherapies for HCC. OV therapy has been shown to increase NK infiltration within the TME, suppressing HCC and reducing recurrence^[70]^. Below we highlight both clinical and pre-clinical studies which have investigated OV therapy for the treatment of HCC.

## Oncolytic Virus Therapy in HCC

The most common OV trials have been in gastrointestinal malignancies (76 studies involving 577 patients) and melanoma (30 studies involving 1000 patients). There have been 6 registered trials relating to liver cancer [Table 1]^[71]^. JX-594, dl1520, H101 and VSV-hIFN-β have all been tested in HCC related clinical trials and developed to exhibit pan-cancer specificity by targeting common molecular abnormalities. Examples of this include TP53 loss, overexpression of the apoptosis inhibitor, survivin and an oncolytic measles virus engineered to target CD133 positive cells, a marker which in the liver, is only expressed on tumour cells^[72]^.

### JX-594 (Pexa Vec)

JX-594 from the Wyeth strain of vaccinia virus, has been genetically modified to encode thymidine kinase (to increase tumour specificity whilst inactivating it), GM-CSF (as an immune modulator) and β-Galactosidase. GM-CSF is a cytokine secreted by multiple cell types inducing proliferation, differentiation and recruitment of APCs, such as macrophages and dendritic cells. JX-594 has the capacity to induce virus-replication dependant oncolysis and to focus the immune response against the tumour^[73]^. In a randomised dose-finding trial (NCT00554372), patients with advanced HCC in the high dose arm had superior overall survival compared to the low dose arm (14.1 months *vs*. 6.7 months respectively, *n* = 30)^[74]^.

One hundred twenty-nine patients with advanced HCC took part in the randomised phase IIb TRAVERSE trial, investigating the use of JX-594 following treatment failure with sorafenib^[75^^]^. Participants were either randomised to JX-594 and best supportive care (BSC) or BSC alone. Unfortunately, there was no improvement in overall survival seen, although significant study limitations were present such as a high dropout rate (63%) in the BSC only arm. Possible reasons limiting the success of JX-594 include studies showing a high prevalence of neutralising antibodies after 4 weeks of treatment^[76]^. However, one positive outcome of the study was the confirmation of viral replication through measurement of β-galactosidase and the development of T cell responses to tumour-specific antigens such as AFP, MAGE-A1/A3/A4 and survivin by ELISPOT. PHOCUS, a phase 3 study (NCT02562755), studied Pexa Vec followed by Sorafenib *vs*. Sorafenib alone in advanced HCC without previous systemic therapy. Unfortunately, an interim analysis was performed, which recommended the closure of the study due to futility, as it was thought unlikely to meet its primary objective. The role of JX-594 in combination therapy is now being explored further in a phase 2 study as a 1st line therapy alongside nivolumab in advanced HCC (NCT03071094). This is a logical combination of therapies that boosts cytotoxic T cell responses using JX-594 whilst simultaneously preventing their inhibition using nivolumab to block the checkpoint molecule PD1, which is induced on activated T cells.

### VSV-hIFN-β

Vesicular stomatitis virus (VSV) is a non-pathogenic negative-stranded RNA virus and, in this therapeutic, is accompanied by the insertion of an interferon-β gene. Defective IFN response pathways are often present within tumour cells allowing oncoselective VSV replication with efficacy seen in a variety of cancers *in vitro* and *in vivo* ^[77]^. VSV-hIFN-β also possesses the potential to halt immune tolerance and promote a potent anti-cancer immune response through stimulation of CD8^+^ T cells and NK cell activation^[78–80]^. In murine models, possible barriers of VSV relate to premature clearance of the virus, reducing the duration it can exert its oncolytic functions^[81,82]^. A phase 1 trial (NCT01628640) is underway to determine the best dose and side effect profile when used in patients with refractory liver cancer and other advanced solid tumours.

### DL520 (ONYX-015) and H101

ONYX-015 is an Adenovirus Type 5, which can selectively replicate within p53 deficient tumour cells. This enhanced cancer specificity is achieved through the deletion of the E1B gene, which binds P53, to serve as an oncolytic vector^[83]^. A phase 2 trial where 19 patients with a hepatobiliary malignancy (5 patients had HCC) received intralesional ONYX-015. Of the 16 patients assessed for a response, 50% had a more than 50% reduction in tumour markers, 1 patient had a partial response and a further participant had prolonged disease stabilisation^[84]^. A possible disadvantage of this OV is the high prevalence of adenoviral antibodies within the population limiting its efficacy risks of horizontal transmission to contacts who may be immunosuppressed.

H101 (Oncorine) is very similar to ONYX-015 but with an additional deletion within its E3 gene. This alteration allows the virus to escape detection and lysis by NK cells and T cells. Oncorine has been licensed for advanced nasopharyngeal cancer in China in combination with 5-Fluorouracil and cisplatin since 2005^[18]^. Transarterial chemoembolisation in combination with H101 has been explored for patients with unresectable HCC, which demonstrated a marginal improvement in overall survival of 1.2 months only^[85,86]^.

## Pre-Clinical HCC OV Research

Multiple pre-clinical OV models have been constructed to try and specifically target HCC through a variety of mechanisms. HCC-specific viral promoters can be transfected into viral genomes that limit viral transcription to only the HCC cell population. It was illustrated by the insertion of apoptin (apoptosis-inducing protein) into recombinant adenovirus using an alpha-fetoprotein (AFP) promoter^[79]^. The effectiveness of this therapeutic option was limited by variability in the expression of HCC cells secreting AFP^[87]^. Specific microRNA (miRNA) signatures serve as another means for differentiating between HCC and healthy tissue and can be used to attenuate the toxicity of healthy tissue. For instance, mir-122, a microRNA that is highly expressed in the liver, can be significantly downregulated in HCC. Insertion of a mir-122 binding sequence within the 3’ UTR of the E1A transcription cassette into an oncolytic type 5 adenovirus, attenuates viral replication in healthy mir-122 expressing hepatocytes, but not HCCs with low expression of this microRNA^[88]^. Thus, this modification to the OV could help to limit viral toxicity to healthy tissue.

The liver is an immunotolerant organ and has an immunosuppressive TME^[89]^. This tolerance is further exacerbated in HCC with the predominance of immunosuppressive cytokines and a scarcity of stimulatory cytokines, such as interleukin-2 (IL-2) and IFN-γ. However, the liver is rich in NK cells and thus represents a legitimate target for OV therapy, which can stimulate innate and adaptive immune responses. Increased NK cell numbers within HCC have been observed following OV administration^[70]^. Interestingly when NK populations within the HCC decline, so does the anti-cancer effect^[90]^. The idea of engineering pro-inflammatory cytokines to accentuate the NK cell response has been proposed. The use of oncolytic adenoviruses encoding IL-12 and tumour necrosis factor-related apoptosis-inducing ligand (TRAIL) has been investigated in pre-clinical models of human HCC^[70]^. Results demonstrated increased tumour necrosis as well as stimulation of an anti-cancer immune response coupled with an increase in NK cell and APC frequency as well as signs of increased functionality through marked upregulation of interferon-γ. This study also highlighted the capability of the engineered OV to replicate and effectively express the inserted vectors (IL-12 and TRAIL). Immune cell responses can also limit tumour growth through the downregulation of vascular endothelial growth factor, a protein that promotes angiogenesis^[70]^.

The M1 virus, a strain of Getah-like alphavirus, can selectively kill HCC, which is deficient in zinc finger anti-viral protein (ZAP). It is a relevant target as low expression of ZAP is seen in 69% of HCC^[91]^. However, pre-clinical studies showed disappointing efficacy highlighting the potential requirement for combination therapy and additional genetic engineering of the virus to improve efficacy. Inhibitors of valosin-containing protein have been observed to be specific inductors of oncolysis in HCC by promoting stress-induced apoptosis^[92]^. A phase 1 trial (NCT04665362) has now been approved to determine the safety, tolerability & efficacy of the M1 virus combined with an anti-PD-1 (SHR-1201) antibody and Apatinib, a tyrosine kinase inhibitor in patients with advanced HCC.

## Safety and Tolerability

OV therapy has been reported to have favourable tolerability and safety profiles compared to other therapies for advanced HCC. It is likely due to the mechanism of action & specificity of OV therapy. A systematic review of 97 clinical trials involving OV therapy found favourable tolerability profiles with common-treatment related adverse events being of low grade^[12]^. The most frequent adverse event encountered was fever, flu-like symptoms and nausea and vomiting. In contrast, a recent meta-analysis investigating the efficacy and safety of OV therapy showed that from 11 randomised controlled trials, there was a significantly higher incidence of severe adverse events as compared to control subjects^[93]^. When comparing safety with routes of OV administration (intratumoural and intravenous), there was no significant difference in the frequency of adverse events^[12]^.

## Modifying Oncolytic Virus Therapy to Enhance NK Cell Responses

The ability of OVs to simultaneously activate the immune system, lyse tumour cells, and express genes of interest, specifically at the tumour bed, makes them a unique therapeutic approach with strong potential for the treatment of cancers including HCC. Multiple approaches to improve the therapeutic efficacy of OV therapy are ongoing, and below, we discuss both current and potential future approaches to activate NK cells alongside OV therapy.

### Insertion of Transgenes to enhance the Anti-tumour NK Cell Response

**Figure 1:**
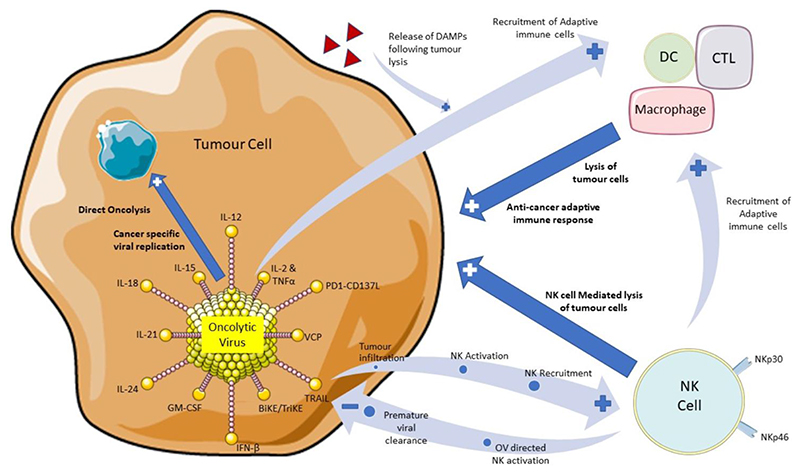
A diagram to illustrate the role of oncolytic virus therapy in stimulating natural killer cells and initiating both an innate and adaptive immune response directed towards tumour cells. Highlighted are approaches under investigation to enhance OV therapeutic efficacy, either by incorporation into the virus or in a combination treatment strategy. Also, highlighted are potential hurdles to OV therapy related to premature viral clearance by NK cells. Illustrations of the OV, tumour cell, NK cell and adaptive immune cells have been used and adapted with permission from *Servier* Medical ART (https://smart.servier.com/). OV: Oncolytic virus; NK: natural killer.

To enhance the recruitment and activation of host anti-tumour immune responses by T cells, DC cells, macrophages and NK cells, gene modification strategies of OVs are being investigated (see Figure 1)^[25]^. Here we will consider how these strategies can specifically modulate NK cell activity and highlight their potential for enhancement of NK mediated anti-tumour responses.

Cytokines such as IL-2, IL-12 and IL-15 drive NK cell activation and proliferation. However, systemic delivery of these cytokines to patients has resulted in unacceptable toxicity. Therefore, localised production of cytokines within the TME produced as part of the viral replication process has the potential to stimulate NK cells, whilst simultaneously limiting the side effects associated with systemic administration. There has been an intense effort in this regard, and a plethora of virus-cytokine constructs have been described, which show promising efficacy in pre-clinical models.

IL-2 promotes NK cell survival, differentiation, cytolytic activity and cytokine production^[94]^ and therefore may enhance NK anti-tumour activity in conjunction with OV therapy. The oncolytic adenovirus TILT-123 co-expresses IL-2 and TNF-α, promotes tumour infiltrating lymphocyte activation^[95]^ and is entering a phase 1 trial for patients with solid tumours (NCT04695327). However, the ability of IL-2 to promote regulatory T cell activity^[96]^ has led to the focus on alternative cytokines, which can specifically increase the activity of cytotoxicity lymphocytes.

IL-12 is a key driver of NK activation against tumour cells through the augmentation of NK cell cytotoxicity and IFN-γ production in particular^[97,98]^. The IL-12 gene has been incorporated into an oncolytic herpes virus (M032), and a Phase 1 Study (NSC 733972) for the treatment of recurrent malignant glioma is currently in the recruitment phase. A vaccinia virus armed with IL-12 was found to significantly prolong survival in surgery-induced metastatic cancer models by controlling lung metastases, with NK cell activation being crucial for efficacy^[99]^. Intraperitoneal injection of autologous tumour cells infected with an oncolytic Maraba MG1 virus expressing IL-12 induced recruitment of NK cells to the peritoneal cavity and caused a substantial reduction in the size of established peritoneal tumours^[50]^. As well as promoting NK cell activity, IL-12 can also inhibit angiogenesis and recruit dendritic cells, macrophages and monocytes to the tumour bed resulting in augmented cytotoxic T cell responses.

IL-15 has an essential role in NK cell proliferation and survival^[98]^ and promotes NK cell anti-tumour effector function via stimulation of IFN-γ, granzyme B and perforin production^[100]^. Various studies have coexpressed IL-15 with oncolytic viruses to utilise the anti-tumour activity of this important cytokine. For example, recombinant myxoma virus (vMyx-IL15Rα-tdTr), expressing an IL15Rα-IL-15 fusion protein, showed enhanced IL-15 bioavailability and stability compared with IL-15 alone^[101]^. vMyx-IL15Rα-tdTr promoted NK cell and T cell tumour infiltration and prolonged survival in a murine model of melanoma^[101]^. An IL-15 and CCL20 (chemokine)-armed oncolytic adenovirus (CRAd-CCL20-IL15) in combination with cytotoxic effector (combination of T cells and NK cells) adoptive therapy showed greater anti-tumour efficacy compared to cytotoxic effector cells or CRAd-CCL20-IL15 alone against colon tumours in a murine model^[102]^. An oncolytic vaccinia virus expressing an IL-15 superagonist, a fusion protein of IL-15 and IL-15Ralpha, showed potent CD8^+^ T cell-dependent but NK independent anti-tumour responses in combination with anti-PD1 antibodies in mice challenged with colon or ovarian tumours^[103]^. This study highlights that the requirement for NK cell activity appears dependent on the type of tumour model and the co-treatment used. A measles virus Schwarz vaccine strain (MeVac) vector encoding an IL-15 superagonist increased NK infiltration into tumours *in vivo*, and MeVac expression of an IL-12 fusion protein was found to promote immune activation and anti-tumour effects^[104]^.

The combination of IL-12, IL-15 and IL-18 induces a memory-like NK cell which is long-lived and has enhanced anti-tumour effects^[105]^. This approach has a strong potential for activating NK cells in combination with OV. In HCC, for example, this combination enhanced NK cell cytotoxicity against liver cancer-derived cell lines and resulted in tumour suppression in a spontaneously arising mouse model of HCC^[106]^. Although this specific combination of cytokines has not yet been used in combination with OV therapies, an oncolytic adenovirus co-expressing IL-12 and IL-18 showed enhanced anti-tumour effects in a murine melanoma model compared to expression of either cytokine alone^[107]^. The addition of IL-15 to this combination may thus further augment NK cell anti-tumour responses.

IL-21 is related to IL-2 and IL-15 as part of the common gamma-chain family of cytokines and has a key role in modulating NK cell activation, maturation and proliferation^[108]^. Insertion of the IL-21 gene into a modified vaccinia virus Tian Tan strain promoted the recruitment of immune effector cells to the tumour bed, reduced regulatory T cell numbers and synergised with CAR-T and invariant natural killer cell following intratumoural injection of the OV^[109]^. Notably, an abscopal effect was also evident, whereby distant non-injected tumour growth was inhibited, and this was also associated with increased numbers of immune effectors, including NK cells within the distant tumours^[109]^. Interestingly oncolytic adenovirus armed with CCL5 and IL-15 enhanced CAR-T recruitment to tumour sites and cytotoxicity in an *in vivo* model of neuroblastoma^[110]^. Thus, although the focus of this work was on studying anti-tumour CAR-T cell responses, simultaneous activation of NK cells is also likely given that both CCL5 and IL-15 are key drivers of NK cell recruitment and activation. Therefore, this approach may prove useful either for enhancing endogenous NK cell responses or in a combination approach with CAR-NK cells to promote anti-tumour efficacy.

A key benefit of inserting cytokine transgenes into the viral construct is the limiting or avoiding of systemic cytokine-associated toxicities. However, localized expression of cytokines may also provide a more local potent anti-tumour stimulus. For example, IL-15 produced within the tumour by an oncolytic vesicular stomatitis virus-induced greater anti-tumour compared to systemic IL-15 administration^[111]^.

An alternative approach to enhance host immune responses during OV therapy is through the insertion and co-expression of genes that encode antibodies or antibody fragments targeting immune checkpoints. It allows the production of checkpoint inhibitors directly within the tumour bed, as opposed to the standard method of systemic administration of these agents. One promising example is an engineered oncolytic herpes virus armed with a single-chain antibody fragment against PD1. This agent was associated with increased survival in murine models of glioblastoma^[112]^. In addition, an oncolytic adenovirus expressing a soluble bispecific fusion protein containing the extracellular domains of PD-1 and CD137L at each terminus (Ad5-PC)^[113]^ blocks PD-1 whilst retaining the stimulating activity of CD137L. This OV was found to increase IFN-γ production in the TME and significantly prolong survival in a murine subcutaneous HCC tumour model. Interestingly, simultaneous injection of an oncolytic adenovirus and a helper-dependent adenovirus expressing a PD-L1 blocking mini-antibody in combination with HER2 targeting CAR T cells, produced superior anti-tumour efficacy in murine models of solid tumours compared to using these therapeutics as single agents^[114]^. These approaches were designed to enhance anti-tumour T cell responses. However, because NK cells have recently been shown to play a key role in PD1/PDL1 checkpoint therapy^[115]^, further investigation into the contribution of NK cells in this setting is warranted. In addition, other checkpoint molecules may also be immunotherapy targets. For instance, blocking the inhibitory receptor NKG2A unleashes both NK cells and T cells, and the anti-NKG2A antibody Monalizumab promoted anti-tumour responses in a recent clinical trial^[116]^. Blockade of NKG2A in combination with OV may therefore provide a promising strategy to simultaneously target both T cells and NK cells.

OVs engineered to express genes encoding bi-specific T cell engager (BiTE) or tri-specific T cell engager (TriTE) to specifically activate T cells have been published in a number of studies. For example, an oncolytic measles virus encoding a BiTE targeting the tumour antigen CEA prolonged survival in a patient-derived colorectal carcinoma xenograft model^[117]^. Analogous to BITEs and TRITEs are the bi-specific killer engager (BiKE) and tri-specific killer engagers (TriKE) which activate NK cells via CD16 and, more recently NKp46^[118]^. The impressive therapeutic efficacy of BiKE and TriKE seen in haematological malignancies indicates that this could be a promising novel approach to enhance OV therapy in a similar manner used for T cell activation. It will be interesting to assess the combination of OV and BiKE/TriKE mediated activation of NK cells against HCC.

Insertion of multiple genes into viral constructs and the subsequent localised expression at the tumour bed of antibodies, cytokines, chemokines, bi-specific antibodies or membrane-bound ligands of choice hold great potential for overcoming the immune-suppressive environment. Four different genes are able to be simultaneously expressed by NG-641, a modified adenovirus that is currently in phase 1 clinical trials (NCT04053283). Expression of multiple genes has to date largely focused on augmentation of T cell activation. However, NK cells are also present within tumours, and their number correlates with the outcome and promotes adaptive immunity^[6,119]^. Thus, the expression of genes targeting NK cells could generate a broader local cellular immune response whilst simultaneously reducing the toxicities and off-target effects associated with systemic therapies. Key NK cell targets for immunotherapy include stimulation of NKG2D, NKp46 and NKp30.

### Oncolytic virus combined with systemic treatment to enhance NK Function

Antibody-mediated targeting of inhibitory receptors (checkpoints) has proved therapeutically useful in a number of cancer settings, with the recent approval of an anti-PDL1 antibody for treating HCC^[120]^. In the context of OV therapy, the combination of checkpoint inhibitors with OVs is a promising approach under investigation. For example, oncolytic vaccinia virus in combination with anti-PDL1 antibodies in a murine colorectal cancer model enhanced CD8^+^ T cell infiltration into tumours and reduced tumour burden more effectively than either treatment alone^[121]^. NK cells express a plethora of inhibitory receptors, including PD-1, which regulate their function and therefore, the effect of anti-PD-1/PDL-1 antibodies in combination with OV therapy may augment NK cell recruitment and activation during checkpoint therapy^[115]^. Additionally, in a model of ovarian cancer, NK cells promoted the activity of two different oncolytic adenovirus when combined with blockade of the inhibitory receptor TIGIT^[40]^. In addition, localised treatment with oncolytic Newcastle disease virus can induce tumour infiltration with NK cells and T cells into distant tumours and sensitise distant tumours to systemic checkpoint blockade (anti-CTLA-4) therapy ^[122]^.

Histone deacetylases (HDAC) inhibitors are known to increase ligands for the key NK cell activating receptor NKG2D. The combination of OV with HDAC inhibitors is another promising combination strategy to target the anti-tumour activity of NK cells^[123,124]^. Pre-clinical studies in HCC identified that HDAC inhibition in combination with oncolytic measles vaccine virus resulted in enhanced primary infections of the OV, increased apoptosis of HCC cell lines, and importantly, there was no loss of OV replication^[125]^.

CAR-T cell therapies have recently gained approval for the treatment of B-cell acute lymphoblastic leukaemia and are currently being assessed in clinical trials for the treatment of HCC. Although CAR-NK cells lag behind CAR-T cells in clinical development, they have been shown to be safe and efficacious in cancer patients^[126]^ and are under investigation for use against HCC^[127]^. Combination of OV therapies with CAR-T cells have shown efficacy in pre-clinical models; for example, an oncolytic adenovirus armed with a PDL1 blocking mini-antibody enhanced the anti-tumour efficacy of HER2 specific CAR-T cells in murine models of solid tumours^[114]^. In addition, EGFR specific CAR-NK combined with oncolytic HSV-1 prolonged survival in an *in vivo* model of metastatic breast cancer compared to either treatment alone^[128]^. An alternative approach to increase NK cell infiltration into tumours has been achieved via lentivirus-mediated upregulation of CCR5 in engineered NK cells in combination with a CCL5 armed vaccinia virus. This combination led to enhanced tumour infiltration of NK cells and prolonged survival in an *in vivo* colon tumour model compared to either treatment alone^[129]^.

Surgery is a viable therapeutic option for early (BCLC stage A) HCC with a potential for cure. However, this can be associated with NK cell dysfunction and subsequent tumour relapse^[130]^. Restoration of NK cell function may be important in this setting to restore anti-tumour immunity. Intriguingly, perioperative administration of oncolytic parapoxvirus ovis (ORFV) and vaccinia virus was associated with increased NK cell activity and a reduction in post-operative metastases^[131]^. It highlights a novel role of OV therapy in restoring innate immunity in patients.

The challenge for the future development of NK cell-targeted therapies in combination with OV therapy will be to maximise the stimulation of anti-tumour functions of NK cells whilst minimising the anti-viral NK cell immune response, which is detrimental to OV persistence and spread. It should be carefully examined for each OV and disease prior to clinical trials to ensure the optimal benefit is provided to patients.

## Conclusions

There is a high unmet clinical need for new therapies in HCC, and the promising data on OV therapy gained from various solid tumour models highlights the potential for OV therapy to improve responses in this setting. NK cells directly kill OV infected and non-infected tumour cells and importantly promote adaptive immunity, which is crucial for sustained cancer regression. Activation of NK cells during OV therapy is important in order to promote a strong and sustained anti-tumour response in combination with strategies to enhance T cell activation. However, the timing of NK cell activation alongside OV therapy must be carefully optimised to avoid detrimental OV clearance by NK cells and to maximise their therapeutic benefit. Further work is now required to define optimal OV and NK cell combination strategies to improve the outcome of patients with HCC.

## Figures and Tables

**Table 1:** HCC specific clinical trials, past and present

Virus	Function of virus	Limitations	Intervention	Study design	Outcome
JX-594 (Recombinant vaccinia)	Insertion of GM-CSF & LacZ gene into thymidine kinase: Increased tumour selectivity GM-CSF stimulates anti-tumoral immunity Compromises tumour blood supply	Cannot be used concurrently with sorafenib due to the latter’s inhibition of viral replication^[79]^ Neutralising antibodies in 50% patients 4 weeks after commencing treatment^[73]^	JX-594 10^8^ plaque-forming units (pfu), 3 × 10^8^ pfu, 10^9^ pfu or 3 × 10^9^ pfu	Phase I trial evaluating safety and efficacy of intratumoural injection of JX-594 in primary HCC or metastatic liver tumours	Fourteen patients were treated, 3 had partial response 6 had stable disease, and 1 had progressive disease. All had grade 1-3 flu-like symptoms [132]
			JX_9_-594 at 10^8^ pfu and 10^9^ pfu via intratumoural injection	Phase II dose finding clinical trial of JX-594 injected into tumour in unresectable HCC	Median survival 14.1 months in high dose compared to 6.7 months in low dose arm^[74]^
			JX-594 and BSC or BSC alone	(TRAVERSE) Phase IIb randomised trial following the failure of sorafenib in advanced HCC	Median OS of 4.2 months JX-594 and BSC compared to 4.4 months in BSC only^[75]^
			JX-594 then sorafenib *vs*. sorafenib alone	(PHOCUS) Phase III trial in advanced HCC in patients naïve to sorafenib	Trial terminated, failed to meet primary objectives at interim analysis (NCT02562755)
			JX-594 and nivolumab	Phase I/IIa trial to determine safety and efficacy of combination therapy as first-line treatment for advanced HCC	Active but not currently recruiting (NCT03071094)
Adenovirus type 5 (ONYX-015) (deletion of E1B gene)	Disruption of E1B protein: Allows preferential viral replication in tumours with a defective p53 pathway	High prevalence of anti-adenoviral antibodies Concerns regarding horizontal transmission^[84]^	Intralesional injection of ONYX-015 at 6 × 10^9^ pfu or 10^10^ pfu	Phase II trial of unresectable hepatobiliary tumours and intralesional administration of ONYX-015 to determine safety and efficacy	Objective response seen in 1 patient out of 4 patients with HCC^[84]^
Adenovirus type 5 (H101)	Disruption of E1B protein: Allows preferential viral replication in tumours with a defective p53 pathway Gene deletion in E3 region inhibiting host immunity and increasing viral replication within tumour		Transarterial injection of H101 plus TACE *vs*. TACE alone	Case-controlled study of 175 with unresectable HCC with OS and PFS as primary endpoints	OS and PFS in H101 arm were 12.8 and 10.49 months respectively and in the TACE alone arm was 11.6 and 9.72 months respectively^[86]^
Vesicular stomatitis virus (VSV) (recombinant VSV expressing IFN-β)	Oncoselective cytotoxicity through defective interferon response pathway in cancer cells	Premature clearance of virus, limited through combination embolisation therapy in murine model^[81,82]^	VSV-IFN-β via intratumoural injection	Phase trial (NCT01628640) to determine safety and efficacy in refractory HCC and other solid tumours	Active but not recruiting
M1 virus (alphavirus)	Induces apoptosis in zinc-finger anti-viral protein (ZAP) deficient cancer cells Sensitise refractory cancer through T cell recruitment and upregulation of PD-L1^[133]^	Anti-tumour activity dependant on cancer cell lines and degree of ZAP deficiency^[92]^	M1 oncolytic virus plus anti-PD-1 antibody and Apatinib	Single-arm open-label phase 1 trial to determine safety and efficacy in patients with advanced HCC	Not started recruiting yet (NCT04665362)

TACE: Transarterial chemoembolization; HCC: hepatocellular carcinoma; BSC: best supportive care; OS: overall survival; PFS: progression-free survival.

## Data Availability

Not applicable.
